# Charting progress in learning health systems: A systematic review of 5 years of definitions, models, and frameworks

**DOI:** 10.1002/lrh2.70006

**Published:** 2025-02-27

**Authors:** Louise A. Ellis, Georgia Fisher, Maree Saba, Genevieve Dammery, Kate Churruca, Samantha Spanos, Carolynn L. Smith, Bianca Forrester, Yvonne Zurynski, Jeffrey Braithwaite

**Affiliations:** ^1^ Centre for Healthcare Resilience and Implementation Science, Australian Institute of Health Innovation Macquarie University Sydney New South Wales Australia; ^2^ Western Victoria Primary Health Network Geelong Victoria Australia; ^3^ University of Melbourne Melbourne Victoria Australia

**Keywords:** definitions, frameworks, learning health systems, learning healthcare systems, systematic review, theory

## Abstract

**Introduction:**

Since being introduced by the Institute of Medicine (IoM) in 2007, the learning health system (LHS) concept has gained traction as a promising solution for achieving systems‐level healthcare transformation. This review of the LHS literature consolidates current understanding of LHS definitions, models, frameworks, and underlying theory, relative to their initial conceptualization by the IoM.

**Methods:**

Three academic databases (PubMed, Embase, and Scopus) were searched for peer‐reviewed literature between 2018 and 2023. Articles were included that explicitly focused on LHSs and described an LHS definition, model, or framework. Extracted article information included key article characteristics and article type; LHS definition(s) and their reference(s); components of LHS models or frameworks; and any reported theories underpinning LHS models or frameworks. Extracted data were examined using thematic and visual network analyses, and practical examples of how the domains of an LHS can be actualized in health settings were synthesized.

**Results:**

The majority of the 226 included articles were nonempirical (47%) and originated from high‐income countries (97%), with a significant portion from the United States (62%). A third of articles described an LHS implemented in a real‐world setting (35%). A significant majority (82%) provided a definition of an LHS, with key concepts centering around “knowledge to practice,” “workplace culture,” and “informatics.” Over half of included articles described an LHS model or framework. From thematic deductive‐inductive coding of 145 LHS models and frameworks, most identified aspects related to Science and Informatics (83%) and a Continuous Learning Culture (81%), with the most prevalent sub‐domain being Supportive System Competencies (76%). Implementation science was the most used theory to underpin existing models and frameworks.

**Conclusions:**

By dissecting LHS definitions, models, and frameworks, we present an integrated framework that can serve as a useful tool for LHS researchers, clinicians, and policymakers working to improve health system performance and outcomes.

## 1 INTRODUCTION

Internationally, healthcare systems are facing common challenges, including aging populations, rising chronic disease prevalence, increasing costs associated with new medical technologies, and growing expectations of healthcare consumers.^1^, ^2^, ^3^, ^4^ Even in the most developed countries, less than two‐thirds of healthcare is delivered in line with evidence‐based guidelines, and one‐tenth of care is associated with iatrogenic harm or adverse events.^5^ Innovation to establish health systems that deliver high‐quality and sustainable care is critical; learning health systems (LHSs) have been proposed as a promising solution to bring about the required systems‐level transformation of healthcare,^5^, ^6^ by building a system capable of “continuous self‐study and improvement.”^7^

Conceptually, LHSs were first formally discussed in 2007 at an Institute of Medicine (IoM, now the National Academy of Medicine) Roundtable on Evidence‐Based Medicine.^8^ Since then, the IoM has advanced LHS concepts through a series of reports.^9^, ^10^, ^11^ In 2013, the IoM defined an LHS as one where “science, informatics, incentives and culture are aligned for continuous improvement and innovation, with best practices seamlessly embedded in the delivery process, patients and families active participants in all elements, and new knowledge captured as an integral by‐product of the delivery experience.”^9^ Alongside this definition, the IoM presented a framework identifying four key interrelated LHS dimensions including: (1) Science and Informatics, that provide real‐time access to knowledge and digitally capture care delivery; (2) Patient–Clinician Partnerships, where patients are engaged, empowered partners in care; (3) Incentives, that reward high‐value care and transparency; and (4) a Continuous Learning Culture, that is supported by the system and its leaders.^9^ The IoM definition and framework are intentionally broad and aspirational, enabling adaptation to various contexts.^8^

**FIGURE 1:**
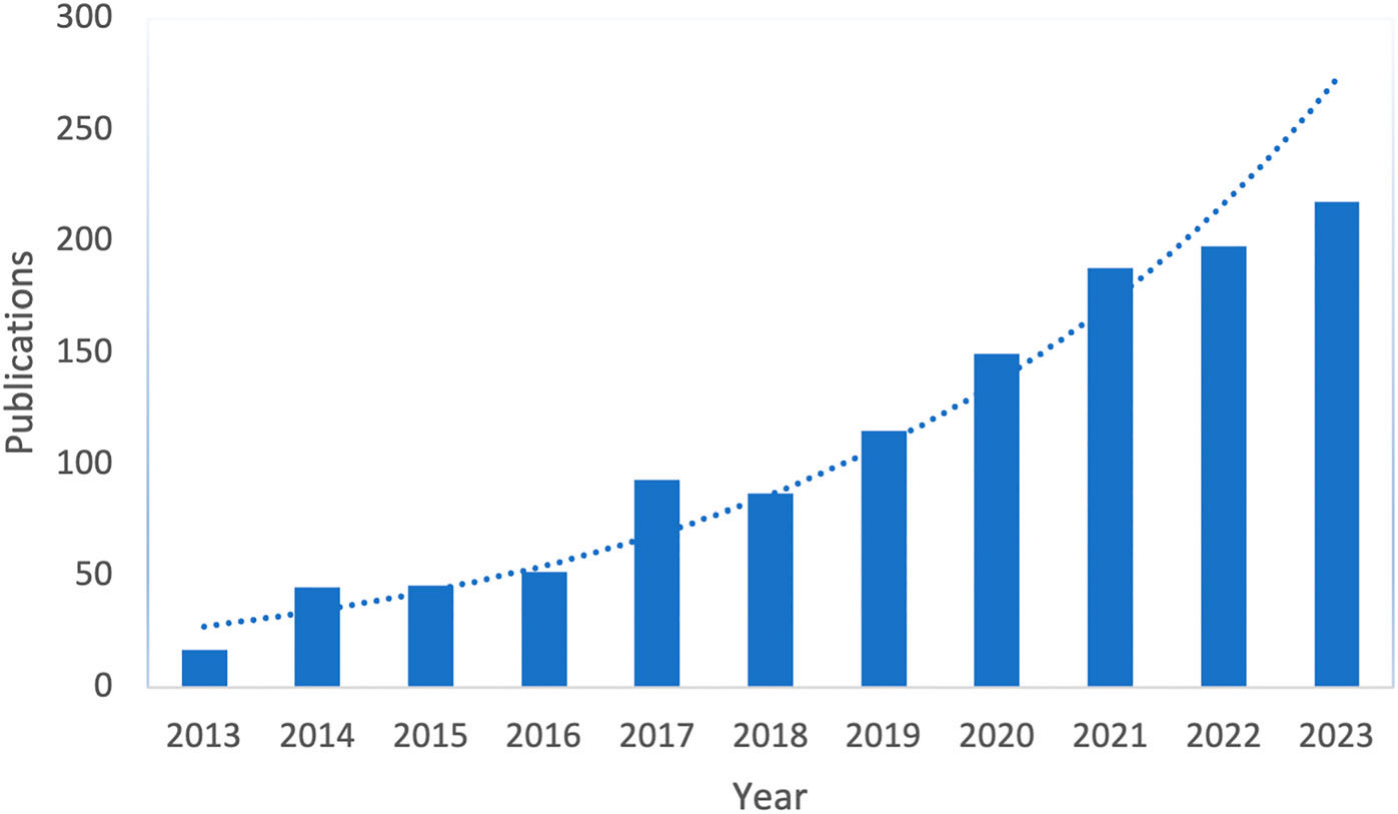
Increase in publications on learning health system over time, 2013–2023 (generated using data from PubMed on publications returned using the search term “learning health system” OR “learning health care system”). The raw count of search hits mentioning these terms is plotted by year. Dotted line: exponential trendline. LHS, learning health system.

Interest in the LHS concept has steadily increased, as evidenced by the continued growth in the number of publications on LHS since 2013 (see Figure 1), including several reviews,^12^, ^13^, ^14^, ^15^, ^16^, ^17^ and the emergence of a dedicated journal, *Learning Health Systems*, in 2017.^18^ There has been a shift toward transforming the concept of LHS from aspiration into reality, with a number of empirical applications more recently being identified,^17^ and a growing interest in policy considerations.^13^ Enthusiasm for LHSs is also reflected in the “panoply of diverse activities, tools, and principles” now associated with the LHS concept.^19^ In 2020, Zurynski et al. conducted a scoping review of the LHS literature from 2016 to 2020. In their associated white paper, “Mapping the Learning Health System,”^15^ Zurynski et al. highlighted the diversity of terminologies and definitions utilized to conceptualize LHSs, the abundance of distinct “schematic” models illustrating the functioning of an LHS, and the ongoing need for further development of its underlying theory.^15^ Additionally, from their synthesis of barriers and enablers to LHS development and implementation, Zurynski et al. added a fifth dimension to the IoM framework, Structure and Governance, to encompass the need for policies, regulations, and governance to facilitate the creation and sustainment of an LHS.^15^ Zurynski et al. further noted that a limited number of LHS models directly incorporate established health system theories (e.g., implementation science)^15^; however, this observation was made on the basis of schematic visual models and did not include a systematic synthesis of descriptive frameworks where LHS concepts may be more thoroughly outlined.

### 1.1 The current study

With the growth of interest and research on LHSs, it is timely to examine the published literature to consolidate current understanding of LHS definitions, models and frameworks, and underlying theory. For this systematic review, a framework was defined as a “set of concepts, and the suggestions of the way that these concepts are integrated,” and a model was defined as “narrower in scope than a framework [where] concepts are well defined, and the relationships between them are specific.”^20^ This review was designed to answer three key research questions:

1. How are LHSs currently being defined, what common concepts do these definitions share and what is the relationship among these concepts?
2. In relation to the modified IoM framework, how are models and frameworks of LHSs currently being conceptualized, what common components do they share and what is the relationship among these components?
3. What elements of existing theory are incorporated into LHS models and frameworks?

Extracted data were examined using thematic and visual network analyses. Visual network analysis approaches are increasingly being recognized as a valuable complement to literature reviews and bibliometric analyses, offering effective and efficient tools for synthesizing the extensive bodies of scientific literature.^21^, ^22^, ^23^, ^24^, ^25^

## 2 METHODS

This systematic review followed the Preferred Reporting Items for Systematic Reviews and Meta‐Analyses (PRISMA) guidelines^26^ (see Appendix 1). The review protocol was preregistered on Open Science Framework: https://doi.org/10.17605/OSF.IO/S7YHM.

### 2.1 Search strategy

Three academic databases (PubMed, Embase, and Scopus) were searched from January 1, 2018 to December 31, 2023 (i.e., the last 6 years, which that were marked by a pronounced rise in LHS publications). The search combined the term “learning health* system*” with terms associated with definitions models and frameworks (see Appendix 2 for full search strategy).

### 2.2 Inclusion and exclusion criteria

Publications were included if they: (1) had an explicit focus on LHSs; (2) described a definition, model, or framework of an LHS; and (3) were peer‐reviewed articles published in English. Publications were excluded if they: did not have an explicit focus on LHSs; did not describe a definition, model, or framework of an LHS; were book chapters or conference proceedings; or the full text was not published in English.

### 2.3 Eligibility screening

Reference details (including abstracts) were downloaded into the reference management software Endnote 20 and then exported to Rayyan QCRI^27^ for title and abstract screening. Five reviewers (GF, RBP, MS, GD, LAE) screened the titles/abstracts to determine their inclusion against the criteria, with 20% of titles/abstracts being blind screened by the entire review team to ensure consistent application of inclusion and exclusion criteria. Raw percent agreement for the 20% blinded title/abstract screening was 85%, and Fleiss Kappa was 0.77, which is considered an acceptable level of agreement.^26^ Included articles from the title/abstract screening underwent a full‐text review by the five reviewers (GF, RBP, MS, GD, LAE). As a final step, RBP screened all articles excluded at both the title/abstract and full‐text stages to confirm the decision to exclude and resolved any conflicts with the wider review team. Regular meetings were also held among the review team to ensure consistency of article inclusion.

### 2.4 Data extraction

Data from included articles were extracted into a custom data extraction form using REDCap software.^28^ The five members of the review team (GF, RBP, MS, GD, LAE) piloted the data extraction form on a subset of five articles to assess the consistency of data extraction and the usability of the form. After minor modifications were made to the form, the remaining articles were distributed among the five reviewers for full‐text data extraction. All extracted data were checked by another reviewer for correctness and completeness, and any disagreements were resolved through discussion.

Key information extracted included: article characteristics (i.e., journal, country of residence of first author); article type (empirical study, structured review, or nonempirical article); LHS implementation stage (implemented or hypothetical/theoretical); LHS definition (including first five reference/s provided); LHS models or frameworks (listing their components and capturing any images provided); and any theories that were used to underpin models or frameworks of LHSs (e.g., implementation science, complexity science, learning theory). For empirical studies, information on study design (i.e., quantitative, qualitative, mixed methods, or case study) was extracted and, for nonempirical studies, the subtype of the article was extracted (e.g., commentary, narrative review, case report). For studies that described an implemented LHS, the LHS health setting (e.g., hospital network, hospital, primary care, aged care, community service) was also extracted.

### 2.5 Quality assessment

To assess the scope and quality of included publications, four quality appraisal tools were used, depending on publication type: (1) Mixed Methods Appraisal Tool (MMAT^29^), (2) Scale for the Assessment of Narrative Review Articles (SANRA^30^), (3) Joanna Briggs Institute (JBI) critical appraisal checklist for systematic reviews and research synthesis,^31^ and (4) the JBI critical appraisal checklist for commentaries and opinion pieces. Five authors (GF, RBP, MS, GD, LAE) each assessed the quality of a portion of the included studies. The quality assessment of each study was checked by another reviewer for correctness and completeness. To make an overall assessment of the quality of each included article across the different quality appraisal tools, we used the maximum possible score in each tool and calculated the percentage of items that each article satisfied. In accordance with previous research,^32^ articles satisfying less than a third of the criteria were considered low quality, between one and two‐thirds of the criteria were considered moderate quality, and over two‐thirds of the criteria were considered high quality.

### 2.6 Data synthesis and analysis

The characteristics of included articles were summarized descriptively via counts and percentages. The country of the first author was coded by income classification based on World Bank definitions of the gross national income per capita. The three categories were low (<US $1135), middle (US $1136–$13 845), and high (>US $13846) income.^33^

Recognising that a comprehensive image of an LHS model or a detailed framework can contain much finer detail than a one‐ or two‐sentence definition of the concept, we conducted separate analyses of the text of LHS definitions, and the components of models and frameworks. By keeping these analyses separate, we aimed to identify differences in how LHSs are being specifically defined and how they are being conceptualized in models and frameworks. These analyses are described below.

Key terms and phrases from LHS definitions were extracted and narratively synthesized. Phrases were consolidated when the meaning was consistent (e.g., “data to knowledge” and “data are then converted into knowledge”). Counts of the most common LHS definitions, and the references used to cite them were also calculated. A visual network of the key definitional concepts was constructed and analysed for frequency and co‐occurrence using Gephi software, version 10.1.^34^ In such networks, the nodes (circles) are the key concepts identified and the ties (lines) represent co‐occurrence (i.e., concepts used together within an article). The size of each node indicates the number of times a key concept was identified. Degree centrality is a measure of the number of ties connecting to a node. Clusters of similar nodes were identified using the Modularity Class statistic,^35^ with different colors being used to represent different clusters.

The content of extracted LHS models and frameworks was qualitatively coded using deductive‐inductive thematic analysis.^36^ Data were coded deductively according to the modified IoM LHS framework (Table 1), which specifies five domains and eight sub‐domains of an LHS. To this coding framework, we added an “Other” category, which captured components of LHS models and frameworks that did not fit within the modified IoM LHS framework. While other popular and well‐cited LHS frameworks have been tendered (e.g., the framework for Value‐Creating LHSs by Menear et al.)^37^ in this review, we aimed to identify how the original IoM conceptualization of a an LHS has been represented in the recent LHS literature. Four reviewers (GF, GD, MS, LAE) met and collaboratively coded data from 10 included studies in real time. Then, each reviewer coded data for a quarter of the remaining studies. Data in the “Other” category were then inductively analysed using content analysis by a single reviewer (GF). Themes were collectively reviewed and refined by four members of the review team, and the raw percentage of their occurrence was calculated. The percentage of models/frameworks that referenced established theories was calculated, as was the raw frequency of different theories.

**TABLE 1:** Modified Institute of Medicine learning health system framework^9^, ^15^ as applied in this study.

Dimension	Characteristics	Description
Science and informatics	Real‐time access to knowledge	Best available evidence incorporated into clinical decision‐making processes to improve the quality of care and patient safety.
	Digital capture of the care experience	Digital platforms (e.g., EHRs, disease registries, mobile devices) utilized for the real‐time capture, production, and application of knowledge based on best available data.
Patient–Clinician partnerships	Engaged, empowered patients	Patients, families, and caregivers are full partners in a patient‐centered system.
Incentives	Incentives aligned for value	Policies actively encourage ongoing evaluation of care and improvement of processes and support the provision of high‐value care and reduction in wasteful practices. Incentives should be aligned across sectors to benefit health providers, health delivery systems, and patients, to provide better outcomes, improve efficiency, and increase engagement.
	Full transparency	All aspects of care, including safety, quality, processes, costs, and outcomes are recorded and available to stakeholders (patients, health professionals, managers) to improve patient care and decision‐making.
Continuous learning culture	Leadership‐instilled culture of learning	Leaders instil a culture of collaboration and adaptability to support the learning process.
	Supportive system competencies	Staff training, skill building, and support to enable continuous refinement of processes and system improvements is implemented.
Structure and governance	Policies, governance, and regulations	Policies, governance, and regulations aligned to facilitate research, collaboration, and learning

## 3 RESULTS

**FIGURE 2:**
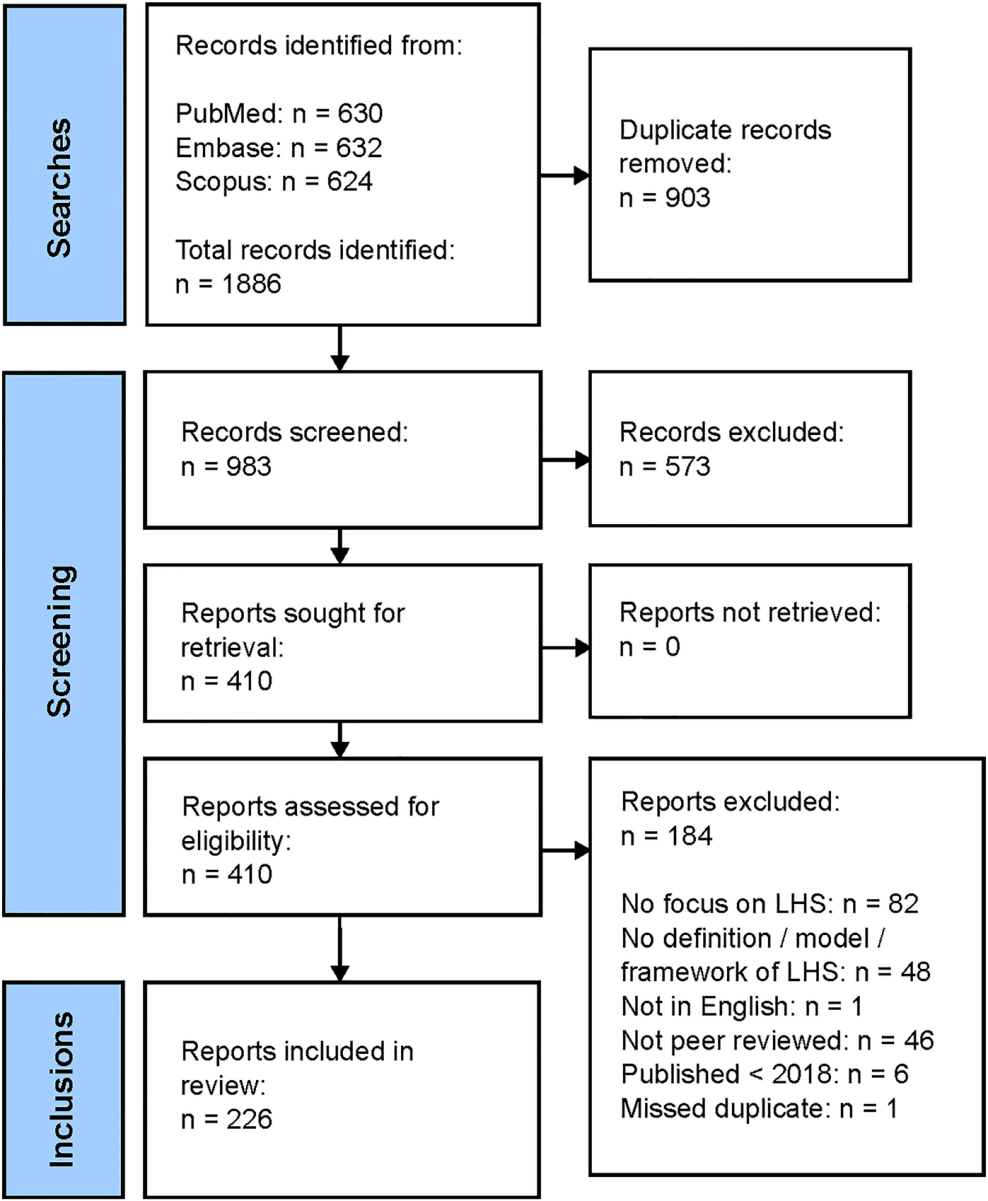
Flow diagram for the study selection process.

The search strategies returned a total of 1886 references, of which there were 983 unique records. Through title and abstract screening, a further 573 records were excluded. The remaining 410 references underwent full‐text extraction, resulting in 226 publications being included in this review. Figure 2 displays the inclusion and exclusion of records at each stage of the screening process.

### 3.1 Publication characteristics

A summary of the key characteristics of the included articles is presented in Table 2 (see Appendix 3 for details of all included articles). Of the 226 included articles, first authors were predominantly from high‐income countries (*n* = 219, 97%), with over half coming from the United States (*n* = 139, 62%), followed by Canada (*n* = 34, 15%), the United Kingdom (*n* = 15, 7%), Australia (*n* = 14, 6%), the Netherlands (*n* = 4, 2%), and Sweden (*n* = 4, 2%). Of the included articles, 107 were nonempirical studies (47%), 98 were empirical articles (42%) and 21 were structured reviews (9%). Close to half of the empirical studies utilized qualitative methods (*n* = 43, 44%), around one‐quarter were mixed methods (*n* = 24, 25%), and the remaining were quantitative (*n* = 21, 21%) or utilized a case study design (*n* = 10, 10%).

**TABLE 2:** Summary of key characteristics of included articles (*N* = 226).

Classification	*N* ^a^	%
Country of corresponding author		
United States	139	61.5
Canada	34	15.0
United Kingdom	15	6.6
Australia	14	6.2
The Netherlands	4	1.8
Sweden	4	1.8
Other	16	7.1
Country income classification		
High	219	96.9
Middle	7	3.1
Article classification		
Empirical study	98	42.4
Nonempirical	107	47.3
Structured review	21	9.3
Empirical study methods		
Qualitative methods	43	43.9
Quantitative methods	21	21.4
Mixed methods	24	24.5
Case study	10	10.2
Nonempirical article type		
Commentary/opinion/perspective	53	49.5
Narrative review	35	32.7
Nonempirical case report	16	15.0
Protocol	3	2.8
Implementation stage		
Implemented	80	35.4
Theoretical	146	64.6
Implemented setting^b^		
Community service^c^	11	13.8
Hospital networks	30	37.5
Hospital/s	22	27.5
Learning network	7	8.8
Primary care	12	15.0
Other	8	10.0

^a^ *N* = number of included articles.

^b^ LHSs were often implemented in multiple settings, therefore, the total number of implemented settings is >80.

^c^ Community services include non‐primary care health services based in the community, such as outpatient clinics, and in‐home care.

Eighty of the included articles (35%) described an LHS that was implemented in a real‐world setting. Of these, the majority were from the United States (*n* = 52, 65%) and were focused on implementation within hospital networks (*n* = 30, 38%), hospitals (*n* = 22, 28%), primary care (*n* = 12, 15%), and/or community services (*n* = 11, 14%). Notably, 10 of the included papers described experiences of creating LHSs in real‐world settings without using empirical methods or reporting data; these articles were classified as nonempirical case reports.

### 3.2 Learning health system definitional citations and key concepts

**FIGURE 3:**
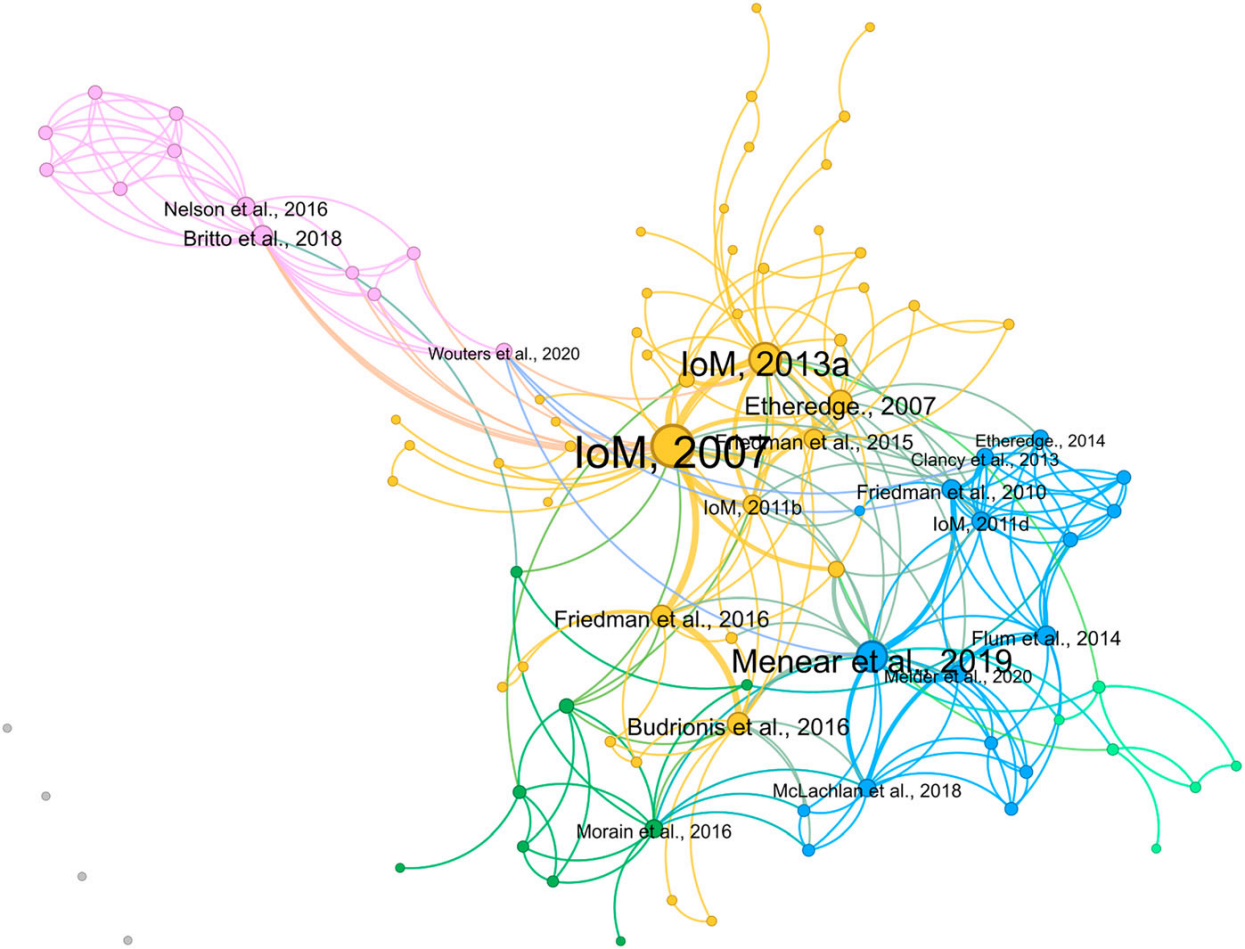
Learning health system definition citation network. Each node in the network represents a definition reference, and the size of each node is indicative of frequency (i.e., larger nodes indicate a higher number of citations). The thickness of the lines (ties) represents the co‐occurrence of citations for a given definition reference. Different colors represent different clusters of citations.

A total of 186 included articles (82%) provided a definition of an LHS. The network analysis of the references used to cite LHS definitions is shown in Figure 3, consisting of 110 nodes and 526 directional ties. In this network, there are five core clusters (i.e., groups of connected nodes), with the main central cluster including the IoM reports; most prominently the IoM 2007 report^8^ and 2013 report^9^ (see Table 3 for the most highly cited references).

**TABLE 3:** Most highly cited references of learning health system definitions (2018–2023).

Source and reference	N
Institute of Medicine^8^	31
Institute of Medicine^9^	25
Menear et al.^37^	24
Etheredge^38^	16
Budrionis et al.^14^	15
Friedman et al.^18^	13
Flum et al.^39^	13

*Note*: *N* = degree centrality (i.e., number of connections to other nodes in the network).

**FIGURE 4:**
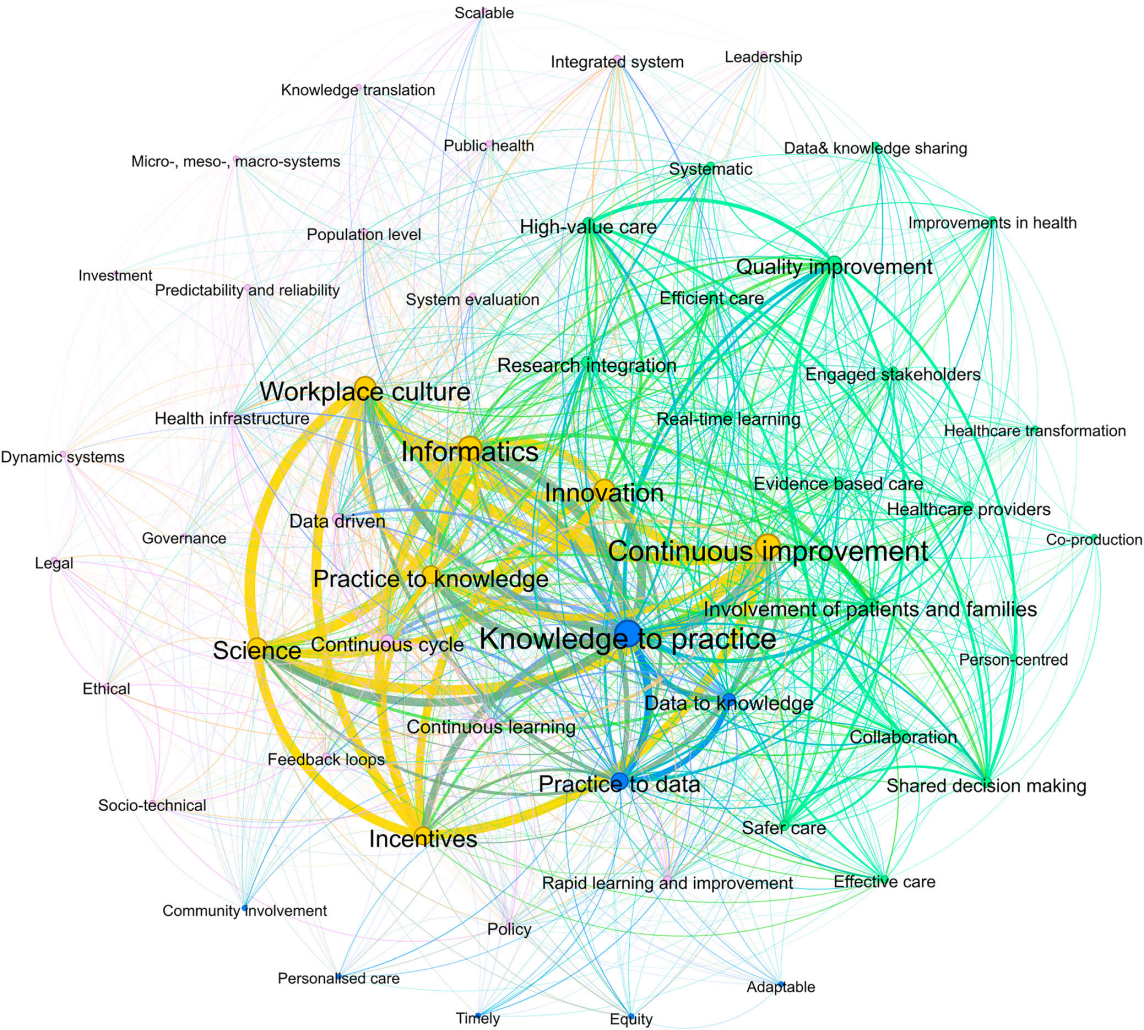
Network analysis of key definition concepts. Each node in the network represents a key concept, and the size of each node is indicative of frequency (i.e., larger nodes indicate a higher number of times the concept was identified). The thickness of the lines (ties) represents the co‐occurrence of key concepts within a given definition. Different colors represent different clusters of definition concepts.

In the text of extracted LHS definitions (*n* = 186 definitions), 56 unique key concepts were identified. Of these, 40 key concepts were identified at least 30 times. A network analysis of the co‐occurrence of key concepts in the text of LHS definitions is shown in Figure 4, which contains four core clusters. The most common key concepts are shown in the central two clusters, which reflect elements of the IoM LHS framework^9^ and of Friedman et al.'s three‐part knowledge‐to‐action cycles: learning cycles convert data to knowledge, apply that knowledge to influence performance, and document changes in performance to generate new data that seeds the next iteration of the cycle.^7^ The most frequently identified concepts synthesized from the text of LHS definitions are summarized in Table 4.

**TABLE 4:** Most prominent key definition concepts (2018–2023).

Key concepts	*N*	Description
Knowledge to practice	53	Generate knowledge and seamlessly apply to practice.
Workplace culture	52	Workplace values, beliefs, and norms.
Informatics	52	A system that leverages the use of digital technologies.
Data driven	51	Harnesses the power of data and analytics to learning.
Continuous improvement	51	Cycles of continuous improvement across the system.
Practice to knowledge	51	Generating knowledge from daily healthcare practices.

*Note*: *N* = degree centrality (i.e., number of connections to other nodes in the network).

### 3.3 LHS models and frameworks

A total of 131 (58%) articles described a model or a framework of an LHS. Some articles reported multiple frameworks; thus, we reviewed the components of 145 LHS models and frameworks across 131 articles. From our deductive coding to the five domains of the modified IoM LHS framework,^9^, ^15^ 121 (83%) models and frameworks incorporated aspects relating to Science and Informatics, 117 (81%) contained elements of a Continuous Learning Culture, 83 (57%) highlighted Patient–Clinician Partnerships, 59 (41%) included the provision of Incentives, and 69 (48%) incorporated aspects of Structure and Governance. From our inductive coding, an additional key LHS domain emerged that we have labeled “Equity and Ethics.” This domain was reported in 36 (24%) models and comprised two sub‐domains: Equity, the provision of care within an LHS that was accessible to all populations, regardless of their demographics; and Ethics, where an LHS was formally based in strong ethical principles. The frequency of each of the six LHS domains is reported in Table 5.

**TABLE 5:** Key domains, sub‐domains, and themes in *n* = 145 LHS models and frameworks described in included studies.

LHS domain	*N* %	LHS sub‐domain	*N* %*	Key themes	Key theme examples	*N* %^#^
Science and informatics	121 83%	Digital capture of the care experience	102 70%	Electronic data capture system	Electronic medical record	72 71%
				Data warehouse/repository	Easy to access, standardized data warehouse	13 13%
		Real time access to data	98 68%	Electronic data provision system	In‐house knowledge database	70 71%
				Clinical decision support tools	Integrated predictive risk modeling	14 14%
Continuous learning culture	117 81%	Supportive system competencies	110 76%	Collaboration and communication	Collaboration across teams at all levels	35 26%
				Knowledge‐based practice	Quality improvement programs	29 26%
				Culture and values	Common learning goals across organization	18 16%
				Continuous improvement	Feedback loops about LHS performance	29 26%
				Education and training	LHS training workshops and competencies	11 10%
		Leadership instilled culture of learning	49 34%	Leadership oversight and support	LHS leadership committee	29 59%
				Multilevel stakeholder leadership	Clinicians, researchers, and policymakers in leadership teams	24 49%
Incentives	59 41%	Incentives aligned for value	39 26%	Financial incentives for staff	Incentivization of value‐based, not volume‐based, care	11 28%
				Organizational incentives	Incentivization of stakeholder involvement in research	23 59%
				Calculation of return on investment	Economic evaluations of LHSs	6 15%
		Full transparency	40 27%	Internal data transparency	Dashboard of LHS performance	28 70%
				External data transparency	Public dissemination of LHS structure and outcomes	17 43%
Patient–Clinician partnerships	83 57%	Engaged empowered patients	83 57%	Patient engagement in LHS	Patient advisory groups, co‐design	56 67%
				Focus on patient‐centered care	Encouragement of shared decision‐making	33 40%
				Capturing patient experience	Patient reported outcome measures	14 17%
Structure and governance	69 48%	Policies, regulations, and governance that support learning	69 48%	Organizational learning strategy	Formalized research agenda	10 14%
				Targeted funding	Financial support for training programs	10 14%
				LHS policies and procedures	Formal LHS workplan	45 65%
				Policy support and oversight	LHS governance committees	38 55%
Equity and ethics	36 25%	Equity	15 10%	Focus on equity of all forms	LHS focus on health equity gaps	15 100%
		Ethics	25 17%	Strong ethical principles	Focus on data protection and privacy	25 100%

*Note*: Percentages of LHS sub‐domains are expressed in relation to the associated LHS domain. Percentages of key themes are expressed in relation to the associated LHS sub‐domain. Examples are provided for each key theme. * Percentages are expressed in relation to the total number of articles that described a framework. ^#^ Percentages are expressed in relation to the total articles that described a framework element in the associated LHS domain.

The LHS sub‐domains were well represented in the models and frameworks described in included studies. The frequencies of their representation, along with key themes and an example from an included model or framework, are presented in Table 5. The most frequently identified sub‐domain related to Supportive System Competencies (*n* = 110, 76%) under Continuous Learning Culture, with prevalent themes including collaboration and communication, knowledge‐based practice, and continuous improvement. The two sub‐domains under Science and Informatics, Digital Capture of the Care Experience, and Real Time Access to Data also featured prominently across models and frameworks.

**FIGURE 5:**
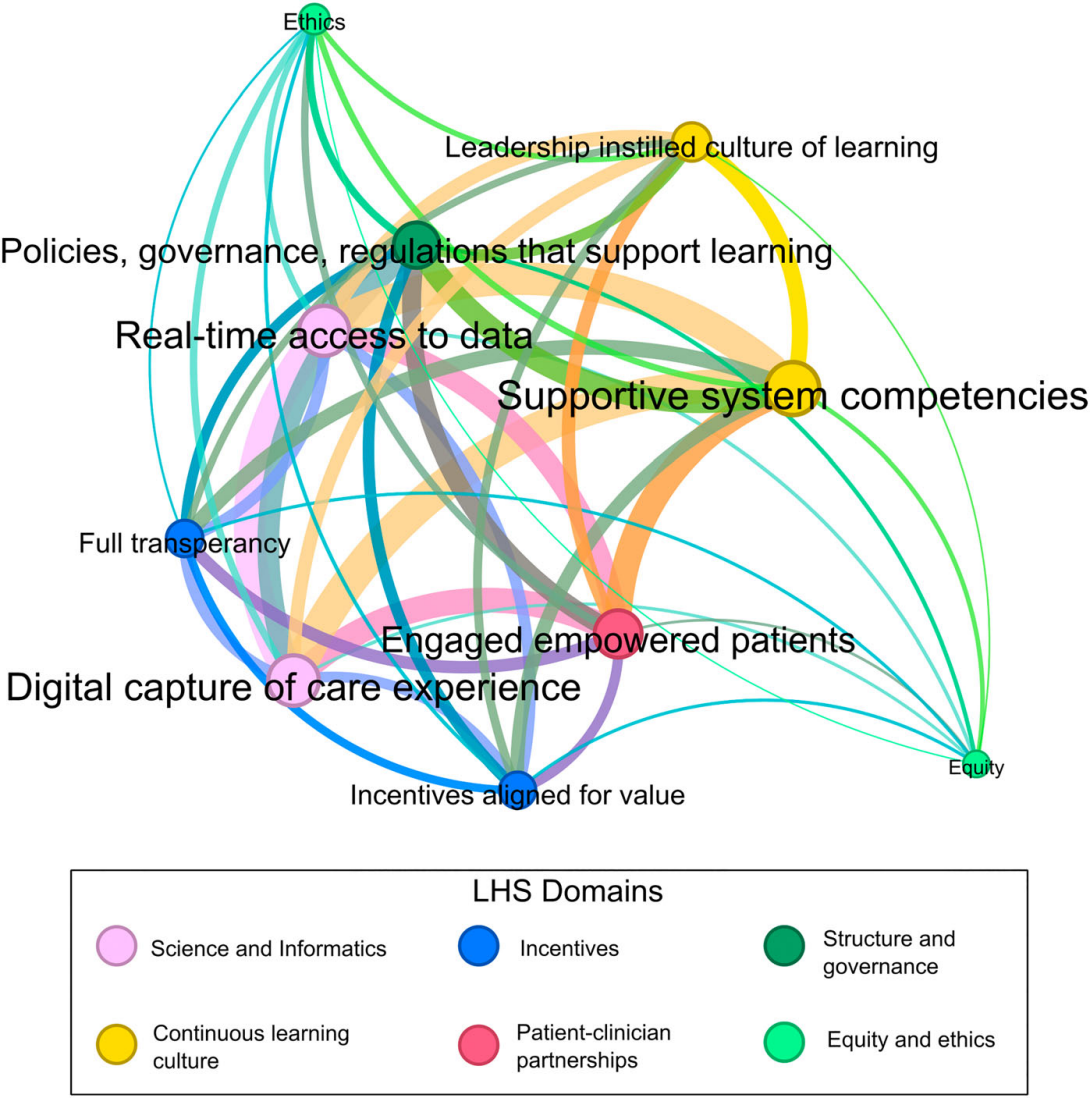
Network map of relationships between sub‐components of learning health system models and frameworks included in this review. Each node in the network represents a sub‐component, and the thickness of the lines between sub‐components represents the frequency with which they co‐occurred. Nodes are colored according to the six LHS domains described in this review, comprising the five domains of the modified IoM Model with the added domain of Equity and Ethics.

The relationship between the 10 LHS sub‐domains was examined through a network analysis of co‐occurring sub‐domains from the 131 articles that described a model or framework (Figure 5). The LHS domains of Science and Informatics, Patient–Clinician Partnerships, and Continuous Learning Culture were most likely to be simultaneously represented in an LHS model or framework. Some sub‐domains were more common than others, for example, Supportive System Competencies, a sub‐domain of a Continuous Learning Culture, were most commonly present alongside Real Time Access to Data, a sub‐domain of Science and Informatics.

### 3.4 Theories in LHS models and frameworks

Of the 145 models and frameworks that were reported in the literature, 65 (45%) were underpinned by established theories or paradigms. The most common theory used was implementation science (*n* = 30, 21%), followed by change theory (*n* = 10, 7%), complexity science (*n* = 10, 7%), and systems theory (*n* = 10, 7%).

### 3.5 Quality assessment of included articles

The MMAT was used to appraise 103 papers, the SANRA for 22 narrative reviews, the JBI Critical Appraisal for Systematic Reviews for 17 papers, and the JBI for 84 commentaries and opinion pieces. Narrative reviews assessed using the SANRA were generally high quality (*n* = 18, 82%), as were systematic reviews assessed using the JBI Systematic Review checklist (*n* = 10, 59%). Similarly, papers assessed using the MMAT were generally high quality (*n* = 81, 79%), as were those assessed with the JBI Text and Opinion checklist (*n* = 84, 100%). Due to the exploratory nature of our analysis, no paper was excluded based on quality.

## 4 DISCUSSION

The literature on LHSs has rapidly developed since their conceptualization in 2007.^8^ Here, we consolidated current understanding of the definitions, models, frameworks, and underlying theory associated with LHSs over the last 6 years (2018 to 2023). Empirical research on LHSs continues to grow,^17^ although there is a predominance of theoretical and conceptual descriptions of LHSs rather than practical applications. The use of established theory to underpin LHSs is developing, particularly in relation to their rapid implementation and evaluation, with increased reference to implementation science in the literature.

Consistent with previous reviews,^17^ most articles were from high‐income countries, particularly the United States—the country where the LHS concept was first coined. Although research focusing on LHS experiences from low‐ and‐middle‐income countries (LMIC) remains limited, there is an increasing divide between the ability of high‐income countries and LMICs in terms of access and use of big data, and rapid advances in technology and research infrastructure, which are key components of LHSs.^40^ Future investigations of LHSs should identify the distinct barriers and enablers to their development and implementation in LMICs, and accordingly, the need for tailored LHS models and frameworks that are designed specifically for these contexts.^40^ By principle, LHSs should be “informed by shared values of universality, equity and serving public health goals,”^40^ however, this review suggests that much work remains to bring the LHS concept to LMIC contexts.

**FIGURE 6:**
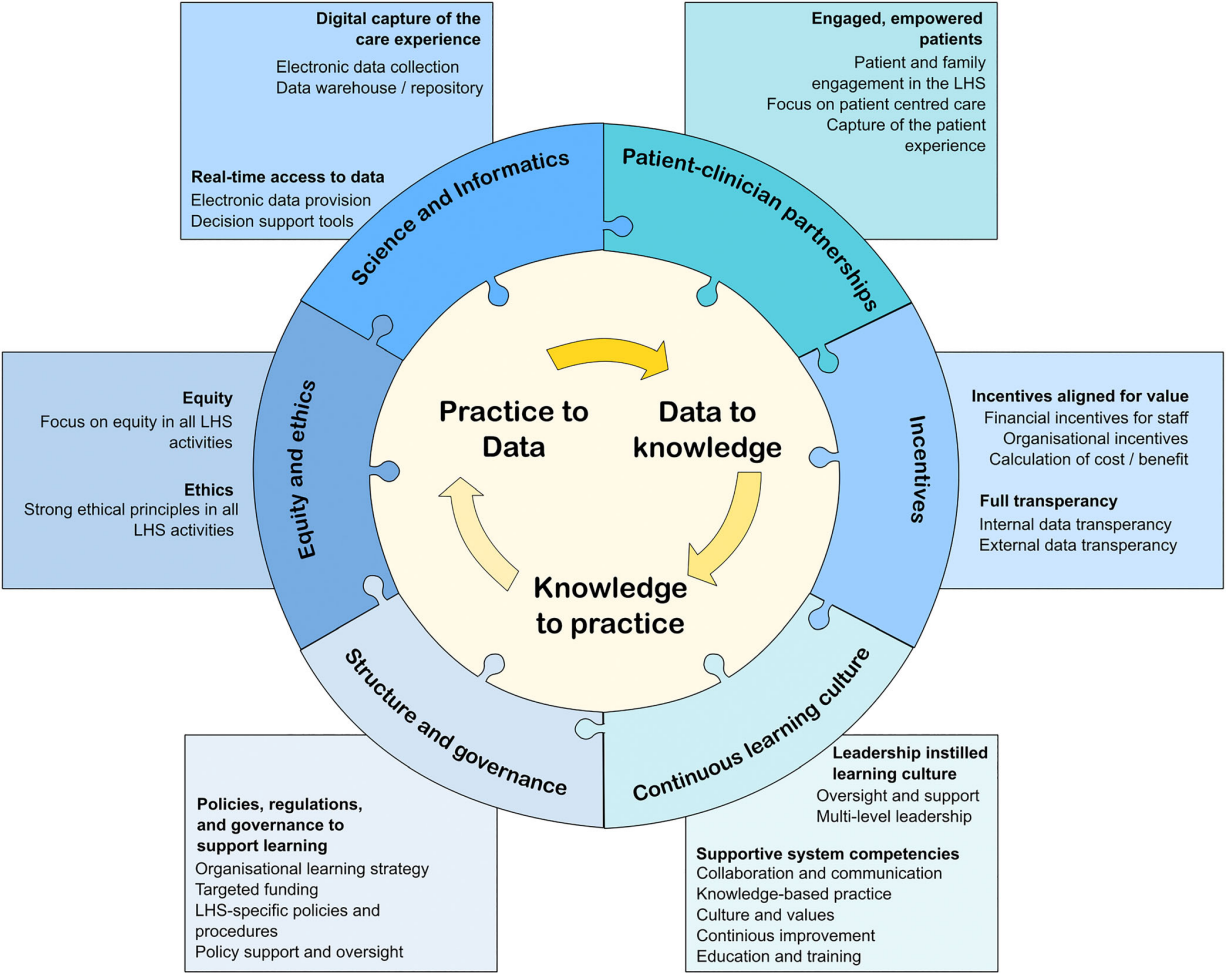
Conceptualization of the learning health system based on the findings of this review.

In accordance with previous reviews,^12^, ^15^ the foundational IoM reports were the most highly cited sources in the definitions of LHSs included here. There has been a convergence around LHS definitions, with key concepts clustering around the 2013 IoM LHS framework^9^, ^41^ and Friedman et al.'s three‐part knowledge‐to‐action cycle.^7^ Based on our thematic deductive‐inductive coding of 145 LHS models and frameworks, and our subsequent network analysis of key LHS domains, we propose an integrated framework to describe LHSs as they are currently being conceptualized and actioned in the literature (Figure 6). In our framework, we have placed Friedman et al.'s three‐part knowledge‐to‐action cycle^7^ (practice to data, data to knowledge, knowledge to practice) in the center, as the central unit of an LHS, surrounded and supported by the six key LHS domains, modified from the IoM framework, that we identified in our review.

As with previous research and the early conceptualization of an LHS by the IoM,^9^ the domains of Science and Informatics, Continuous Learning Culture, Patient–Clinician Partnerships, and Incentives were central to LHSs described in the included publications. The focus of data capture from digital sources (e.g., electronic health records) that are linked to other sources of health data, and the application of predictive modeling or clinical decision support to guide decision‐making, serve as the foundation of an LHS.^42^, ^43^, ^44^ The capture of patient experience data, such as through patient‐reported outcome measures, was also highlighted,^45^, ^46^, ^47^ as was a deliberate focus on patient‐centered care.^19^, ^48^, ^49^ Both internal and external data transparency were key components of Incentives,^50^, ^51^, ^52^ and the use of formal incentives to encourage participation in the LHS was an emerging concept.^53^, ^54^

Structure and Governance was first identified as a key LHS domain in 2020 by Zurynski et al.^15^ Our results confirm the importance of this domain to the conceptualization of an LHS. Aspects of LHSs related to Structure and Governance were typified by formal governance that suits the needs of the system, for example, the Cirrhosis Quality Collaborative (CQC) is a multisite learning health network that is closely affiliated with the American Association for the Study of Liver Diseases (AASLD). The AASLD has recently taken charge of the management of the CQC, so that the learning network is well supported by the experience and resources of a large organization.^55^ A different approach was taken by the Veterans Affairs' Quality Enhancement Research Initiative (QUERI), a national knowledge translation program structured as an LHS, which instead involved multiple enterprise‐wide governance and strategic committees.^56^ While Structure and Governance is essential to incorporate in an LHS, its operationalization should be closely tailored to the needs and goals of each individual LHS.

Another domain that emerged from our synthesis was Equity and Ethics. LHS equity is defined as the elimination of systematic disparities among groups that experience varying levels of social advantage or disadvantage.^57^ Equity was described by Parsons et al.^58^ who identified seven practices to incorporate equity in LHSs: (1) establish equity as a principle, (2) use data to measure equity, (3) ensure leaders have lived experience, (4) co‐produce all aspects of the LHS, (5) redistribute power away from centralized structures, (6) practice a growth mindset, and (7) engage with sectors beyond the traditional healthcare system. For example, the Diabetes Center at Cincinnati Children's Hospital has used clinical registry data to identify variation in clinical measures, emergency department visits, and likelihood of admission for diabetic ketoacidosis (DKA) that depended on a child's race and neighborhood poverty.^59^ They planned to address these variations via multiple, evidence‐based strategies in an LHS structure and have since seen improvements in their cohort of at‐risk patients.^60^ International focus on health equity^61^ means it will be essential for LHSs to learn from such examples and incorporate equity‐based principles into their foundations.^62^

The sub‐domain of Ethics in LHSs was typified by a focus on LHS‐specific ethical frameworks and guidelines, commonly using dedicated LHS institutional review boards and focusing specifically on elements of data privacy and security.^63^, ^64^ For example, Vahidy et al.^65^ described the Houston Methodist COVID‐19 Surveillance and Outcomes Registry (CURATOR), an LHS formed in response to COVID‐19. They established a retrospective research task force with close links to their institutional review board, facilitating rapid ethical approval of data usage for research and learning. Strong ethical principles contribute to multiple domains of an effective LHS, facilitating real‐time access to system data, allowing data to be rapidly converted into knowledge and disseminated widely, and ensuring that data transparency does not breach extant legislation and regulations.^62^ LHSs should thus ensure that their system is underpinned by strong and clear ethical principles, drawing support from organizations and committees that can help define these principles.

In addition to the present review, our research group has conducted several reviews of the structure and function of LHSs based on the IoM model.^15^, ^32^, ^41^, ^66^ Thus, comparing the results provided here with those of previous reviews facilitates a description of how the original IoM conceptualization of an LHS has shifted over the years. As identified in our foundational LHS white paper, which examined LHS frameworks up to May 2020, the IoM domain of Science and Informatics was the most represented, followed closely by Continuous Learning Culture, while Incentives and Patient–Clinician Partnerships were less commonly considered. Here, our results indicate a slight shift in this representation in recent years, with Patient–Clinician Partnership now featuring more prominently in 57% of the included articles. This shift likely reflects the increased focus on both patient‐centered care and the co‐design of healthcare alongside healthcare consumers in the broader contexts of health systems and medical research.^67^

Our research group has also conducted two additional, more focused reviews of LHSs: one reviewing how LHSs are being used to adapt real‐world health systems for future pandemics and climate change,^32^ and another examining LHSs in front‐line care settings (i.e., primary care and emergency departments). The breakdown of IoM domains in these reviews revealed slight differences, suggesting that the utility of each domain, along with the barriers and enablers to achieving them, varies based on the specific context and objectives of the LHS. Indeed, our previous white paper identified that the frequency and type of implementation barriers and enablers varied across LHS domains, for example, a lack of infrastructure, poor interoperability, and poor data quality control were common barriers to the realization of the Science and Informatics domain, while poor alignment between funding for research and learning and funding for health system operation was commonly cited as a barrier to the implementation of Incentives.^15^ Although a comprehensive description of these barriers and enablers was beyond the scope of the current review, they are a critical area for future investigation. Future research should not only examine the barriers and enablers associated with specific domains but also explore how these factors vary across different health system contexts. Additionally, under half of the LHS models and frameworks that we included in this review were underpinned by an established theory, which could reflect either the difficulties of applying theories in this area or simply represent a missed opportunity to strengthen novel models and frameworks by using well‐investigated theoretical principles. A promising area for future research would be to examine the impact of using established theories on the success or rigor of LHS models and frameworks.

A core finding of this review is the rapid proliferation of LHS models and frameworks throughout the evidence base. While the original IoM framework remains a useful method of conceptualizing LHSs, our results show that there is a plethora of alternative ways to achieve the same aim. For example, Allen et al. described the Kaiser Permanente Washington (KWPA) Logic Model of an LHS, a US‐based model divided into Inputs, Outputs, and Outcomes.^45^ Here, elements such as “People and Partnerships” and “Funding” are classified as Inputs, and defined as essential elements for an organization to successfully operate an LHS. These contribute to Outputs, key organization activities and deliverables that add value to care delivery, such as “Data analytics and Evaluation.” Outputs lead to Outcomes, including short‐term and long‐term goals, like “Population Health and Equity.” Conversely, a collaboration of researchers from the United States, United Kingdom, and New Zealand created the Heimdall framework^68^ to characterize and classify LHSs, which makes distinctions between the level at which an LHS operates, for example, “Patient‐Facing LHS,” a “Learning Organization‐based LHS,” and “System‐Based LHS.” Overall, we found that the majority of alternate LHS framework components fit within the modified IoM framework, however, there were naturally some elements that were distinct. Thus, while the modified IoM framework is an important foundation to conceptualizing LHSs, it may not be a “one size fits all” approach. Individuals and organizations that seek to implement LHSs may therefore wish to explore alternate models, particularly those operating in a significantly different health system context to North America from which the IoM framework emerged.

### 4.1 Strengths and limitations

We focused on LHS definitions, models, and frameworks, along with their underlying theory over the last 5 years, examining the breadth and type of changing conceptualizations over time. We included both thematic and novel visual network analyses, a structured method for graphically visualizing complex patterns and themes, which enhanced our understanding of LHS key concepts and the relationships between them. By restricting the review to English‐language journal articles, a significant portion of the work originated from high‐income countries, which represents a limitation of this work. There is a need to include LHS publications in languages other than English for a more comprehensive view of the LHS concept and its global adoption, especially considering the increasing focus on equity in LHS models and frameworks. Another limitation is that most journals impose article processing charges for open access, restricting researchers from lower‐income countries from publishing their work and contributing to further inequities. Further, although the inclusion of three databases is a strength of the study, the elimination of nearly 50% of duplicate articles indicates that future reviews on this topic should consider incorporating less duplicative databases, such as CINAHL.

Additionally, by first using the IoM framework deductively in our analysis, and then inductively synthesizing other LHS components, we acknowledge the potential impact of confirmation bias on our analyses. We note the existence of other prominent LHS frameworks, notably that of Menear et al., who in 2019 proposed a framework for value‐creating LHSs,^37^ which takes a considerably different form from the IoM framework. While the Menear definition is indeed popular (the third most cited definition in our review), our review aimed to track the progress of the original IoM conceptualization of an LHS in the current literature, and we felt that trying to create a hybrid of frameworks in the first instance would unnecessarily complicate the analysis. Future work could replicate this review by deductively analyzing the data according to the Menear framework, as opposed to the IoM framework, and contrast their results against our own.

### 4.2 Conclusion

Research on LHSs continues to develop. Empirical research on LHSs is expanding, although there is still a predominance of theoretical and conceptual descriptions of LHSs rather than practical applications. The use of established theory to underpin LHSs is growing, particularly in relation to their rapid implementation and evaluation, with increased reference to implementation science in the literature. This review consolidates current understanding of definitions, models, and frameworks of LHSs to present a comprehensive framework that can serve as a useful tool for researchers, clinicians, and policymakers working to improve health system performance and outcomes.

## CONFLICT OF INTEREST STATEMENT

The authors declare no competing interests.

## Supporting information


**Appendix 1.** PRISMA checklist.

**Appendix 2.** Search strategies.

**Appendix 3.** Articles included in this study.

## Data Availability

The datasets used and/or analyzed during the current study are available from the corresponding author upon reasonable request.
