# Supplementary material for: Charting progress in learning health systems: A systematic review of 5 years of definitions, models, and frameworks
Source: Learn Health Syst. 2025 Feb 27;9(3):e70006. doi: 10.1002/lrh2.70006 (PMC12264394; doi:10.1002/lrh2.70006)
Supplement: Supplementary file 2 — Appendix 2. Search strategies. [file LRH2-9-e70006-s001.docx]

**Appendix 2: Search strategies**

**PubMed**

("learning health* system*") AND (model* or framework* or theor* or structure or paradigm* or version* or defin* or component*) AND (("2018/01/01"[Date - Publication] : "3000"[Date - Publication]))

**Scopus**

TITLE-ABS-KEY ( "learning health* system*" ) AND (model* or framework* or theor* or structure or paradigm* or version* or defin* or component*) AND ( LIMIT-TO ( PUBYEAR , 2023 ) OR LIMIT-TO ( PUBYEAR , 2022 ) OR LIMIT-TO ( PUBYEAR , 2021 ) OR LIMIT-TO ( PUBYEAR , 2020 ) OR LIMIT-TO ( PUBYEAR , 2019 ) OR LIMIT-TO ( PUBYEAR , 2018 ) )

**Embase**

1 "learning health* system*".mp. or learning health system/

2 conceptual framework/ or framework*.mp.

3 theor*.mp.

4 model*.mp.

5 structure.mp.

6 paradigm*.mp.

7 version.mp.

8 defin*.mp.

9 component*.mp.

10 2 or 3 or 4 or 5 or 6 or 7 or 8 or 9 11 1 and 10

12 Limit 11 to last five years
